# Supplementary material for: Impact of Foliar Application of Amino Acids on Essential Oil Content, Odor Profile, and Flavonoid Content of Different Mint Varieties in Field Conditions
Source: Plants (Basel). 2022 Nov 1;11(21):2938. doi: 10.3390/plants11212938 (PMC9731322; doi:10.3390/plants11212938)
Supplement: Supplementary file 1 [file plants-11-02938-s001.zip › plants-1923797-SI.pdf]

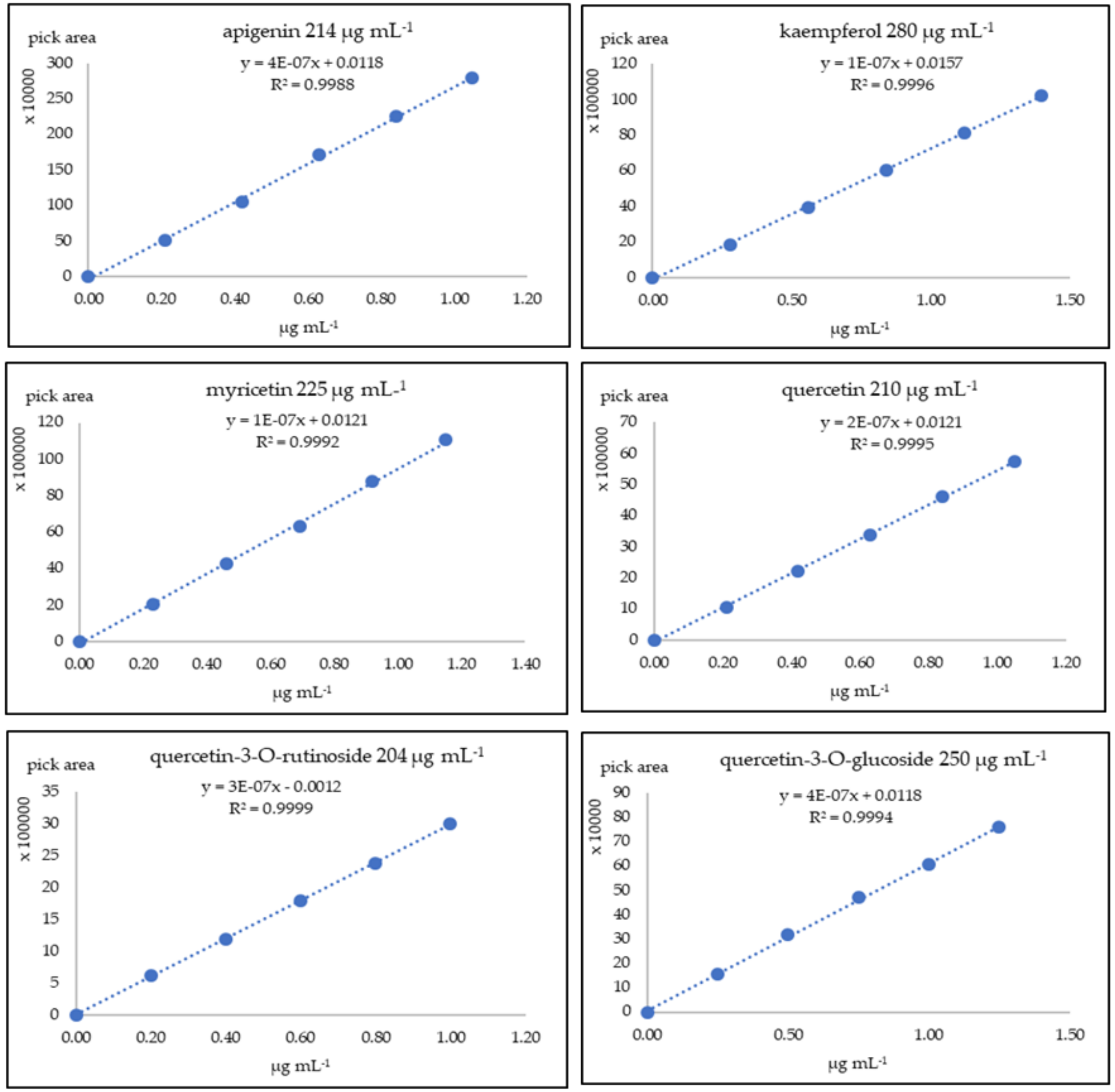

Figure S1. Standard curves for all identified flavonoids in *Mentha* species, detection: 370 nm.

Table S1. Possible compounds identified by the electronic nose in essential oil of different varieties of mints sprayed with amino acids

Serial number	Kovats retention	Possible matches compounds	Chemical group of compounds	Sensory descriptors	*Species/varieties*																																							
					M. spicata **'Moroccan'**								***M. spicata*** **'Crispa'**								***M. piperita*** **'Granada'**								***M. piperita*** **'Swiss'**								***M. piperita*** **'Multimentha'**							
					**Treatment**																																							
					**1**	**2**	**3**	**4**	**5**	**6**	**7**	**8**	**1**	**2**	**3**	**4**	**5**	**6**	**7**	**8**	**1**	**2**	**3**	**4**	**5**	**6**	**7**	**8**	**1**	**2**	**3**	**4**	**5**	**6**	**7**	**8**	**1**	**2**	**3**	**4**	**5**	**6**	**7**	**8**
1	477	Propanone	Ketones	Pungent	-	-	-	+	-	+	-	-	-	-	+	-	-	-	-	-	-	+	+	-	-	-	-	+	-	-	-	-	-	-	-	-	-	-	-	+	+	-	+	-
2	558	Tert-Butylmethylether	Ethers	Pungent	-	-	-	-	-	-	-	+	-	+	-	-	-	-	-	-	-	-	-	+	-	-	-	-	-	-	-	-	-	-	-	-	-	-	-	-	-	-	-	-
3	600	Ethyl acetate	Esters	Anise	-	-	-	-	-	-	-	-	-	-	-	-	+	+	+	+	-	+	+	+	+	-	-	-	-	-	-	-	-	-	-	-	-	-	-	-	-	+	-	+
4	639	Isopropyl acetate	Esters	Fruity	-	-	-	-	-	+	+	+	-	-	-	-	-	-	-	-	-	-	-	-	-	-	-	-	-	-	-	-	-	-	-	-	-	-	-	-	-	+	-	+
5	657	n-butanol	Alcohols	Fruity	+	+	+	+	+	-	+	+	-	-	-	-	+	-	-	-	-	-	-	-	-	-	-	-	-	-	-	-	-	-	-	-	-	-	+	-	-	-	-	-
6	686	Acetoin	Ketones	Butter	-	-	-	-	-	-	-	-	+	+	-	+	+	+	-	+	-	-	-	-	+	-	-	-	+	-	+	+	+	+	-	-	-	-	-	-	-	-	-	-
7	702	Methylbutanoate	Esters	Fruity	-	-	-	-	+	-	-	-	-	-	-	-	-	-	-	-	-	-	-	-	-	-	-	-	-	-	-	-	-	-	-	-	-	-	-	-	-	-	-	-
8	711	Propyl acetate	Esters	Fruity	-	-	-	-	-	-	-	-	-	+	-	-	+	-	-	-	+	-	-	-	+	-	-	-	-	-	-	-	-	-	-	-	-	-	-	-	-	-	-	-
9	733	Isoamyl alcohol	Alcohols	Fruity	-	-	-	+	-	+	-	-	-	+	-	-	-	-	+	+	-	-	-	-	-	-	-	-	-	-	-	-	-	-	-	-	-	-	-	-	+	+	+	-
			Sulfides	Garlic	-	-	-	-	+	-	+	-	+	+	+	+	+	+	-	-	+	+	-	+	-	+	+	-	-	+	+	+	+	+	+	+	+	+	+	+	-	+	-	+
10	797	Butyl acetate	Esters	Fruity	-	-	-	-	-	-	+	-	-	-	-	-	-	-	-	-	-	-	-	-	-	-	-	-	-	-	-	-	-	-	-	-	-	-	-	-	-	-	-	-
11	802	Ethyl butyrate	Esters	Fruity	-	-	+	-	-	-	-	-	-	-	-	-	-	-	-	-	-	-	-	-	-	-	-	-	-	-	-	-	-	-	-	-	-	-	-	-	-	-	-	-
12	820	Butanoic acid	Esters	Butter	-	-	-	-	-	-	-	-	-	-	-	-	-	-	-	+	+	-	-	-	-	-	-	-	-	-	-	-	-	-	-	-	-	-	-	-	-	-	-	-
13	830	Furfural	Aldehyde	Spice	-	-	-	-	+	-	-	-	-	-	+	-	-	-	+	-	-	-	+	-	-	+	-	-	-	-	+	+	-	+	-	+	-	-	-	-	-	-	-	-
14	840	Leaf alcohol	Alcohols	Earthy	-	-	-	+	-	+	-	-	-	-	-	-	-	-	-	-	-	-	-	+	-	-	+	-	-	-	-	-	-	-	-	-	-	-	-	+	-	-	-	-
15	857	2-hexen-ol	Alcohols	Fruity	-	-	-	-	-	-	-	-	+	+	+	+	+	+	-	+	-	-	-	-	-	-	+	-	-	-	-	-	-	-	-	-	-	+	-	+	-	-	-	-
16	878	Isoamyl Acetate	Esters	Fruity	-	-	-	-	-	-	-	-	-	-	-	-	-	-	-	-	-	-	-	-	-	-	-	-	-	-	-	-	-	+	+	+	-	-	-	-	-	-	-	-

17	930	2-Acetyl-1-pyrroline	Ketones	Nutty	-	-	-	-	-	-	-	-	-	-	-	-	-	-	-	-	-	-	-	-	-	-	-	-	-	-	+	-	-	-	-	-	-	-	-	-	-	+	-	-
18	931	Benzaldehyde	Aldehyde	Cherry	-	-	+	-	-	-	-	-	-	-	-	-	-	-	-	-	-	+	+	-	+	+	-	-	-	-	-	-	-	-	-	-	-	-	-	-	-	-	-	-
19	950	Alpha-pinene	Terpenes	Pine	-	-	-	-	-	-	-	-	+	-	-	-	-	-	-	-	-	-	-	-	+	+	-	-	-	-	-	-	-	-	-	-	-	-	-	-	-	-	-	-
20	964	Dimethyl trisulfide	Sulfides	Sulfur	+	+	-	-	-	-	-	-	-	-	-	+	-	-	-	+	-	-	-	-	-	-	-	+	-	-	-	-	+	-	-	+	-	-	-	-	-	-	-	-
21	974	Sabinene	Terpenes	Pine	-	-	+	+	-	+	+	+	-	-	-	-	-	-	-	-	-	-	-	-	-	-	-	-	-	-	-	-	-	-	-	-	-	-	-	-	-	-	-	-
22	989	Ethyl hexanoate	Esters	Fruit	+	+	+	-	+	-	+	+	-	-	-	-	-	-	-	-	-	+	-	-	-	-	-	-	-	-	-	-	-	-	-	-	-	-	-	-	-	-	-	-
23	990	Cumene	Ketones	Plastic	-	-	-	-	-	-	-	-	-	-	-	+	-	+	-	+	-	-	-	-	-	-	-	+	+	+	+	+	+	+	+	+	-	-	+	-	+	+	-	+
24	1008	Alph phellandrene	Terpenes	Mint	-	-	-	-	-	-	-	-	-	-	-	-	-	-	-	-	-	-	-	-	-	-	-	-	+	+	+	+	+	+	+	+	-	-	-	-	-	-	-	-
25	1028	Beta-Phellandrene	Terpenes	Mints	+	+	-	+	+	+	+	+	-	+	+	+	+	+	+	-	-	-	-	-	-	-	-	-	-	-	-	-	-	-	-	-	-	-	-	-	-	-	-	-
26	1035	Limonene	Terpenes	Citrus	+	+	+	+	-	+	+	+	-	-	-	-	-	-	-	-	-	-	-	+	-	-	-	-	-	-	-	-	-	-	-	-	-	-	-	+	-	-	-	-
27	1036	1,8 Cineole	Ethers	Eucalyptus	-	-	-	-	-	-	-	-	-	-	-	-	-	-	-	-	-	-	-	-	+	-	-	-	-	-	-	-	-	-	-	-	-	-	-	-	-	-	-	-
28	1070	1-octanol	Alcohols	Floral	+	+	-	-	-	-	-	-	-	-	-	-	-	-	-	-	-	-	-	-	-	-	-	-	-	-	-	-	-	-	-	-	-	-	-	-	-	-	-	-
29	1084	Terpinolene	Terpenes	Pine	-	-	+	+	+	-	+	+	-	-	-	-	-	-	-	-	-	-	-	-	-	-	-	-	-	-	-	-	+	-	+	-	-	+	-	-	-	-	-	-
30	1097	P-Cymene	Terpenes	Citrus ų Citrus	+	-	-	-	-	-	-	-	-	-	-	-	-	-	-	-	-	-	-	-	-	-	-	-	-	-	-	-	-	-	-	-	-	-	-	-	-	-	-	-
31	1114	Limonene oxide	Terpenes	Fruit	-	-	-	-	-	-	-	-	-	-	-	-	-	+	+	-	-	-	-	-	-	-	-	-	-	-	-	-	-	-	-	-	-	-	-	-	+	-	-	-
32	1207	Trans-Carveol	Alcohols	Caraway	-	-	-	-	-	-	-	-	-	-	+	+	+	-	-	+	-	-	-	-	-	-	-	-	-	-	-	-	-	-	-	-	-	-	-	-	-	-	-	-
33	1224	Nerol	Alcohols	Floral	+	+	-	-	+	+	-	+	-	-	-	-	-	-	-	-	-	-	-	-	-	-	-	-	-	-	-	-	-	-	-	-	-	-	-	-	-	-	-	-
34	1253	Geraniol	Terpenes	Rose	-	-	-	-	-	-	-	-	-	-	-	+	-	+	-	+	-	-	-	-	-	-	-	-	-	-	-	-	-	-	-	-	-	-	-	-	-	-	-	-

35	1225	Citronellol	Terpenes	Citrus	-	-	-	-	-	-	+	-	-	-	-	+	-	-	-	-	-	-	-	-	-	-	-	-	-	-	-	-	-	-	-	-	-	-	-	-	-	-	-	-
36	1257	Cinnamaldehyde	Aldehyde	Spice	-	-	-	-	-	-	-	-	-	-	-	-	-	-	-	-	-	-	-	-	-	-	-	-	-	+	-	-	-	-	-	-	-	-	-	-	-	-	-	-
37	1276	Anethole	Ethers	Anise	-	-	-	-	-	-	-	+	-	-	-	+	-	-	-	-	-	-	-	-	-	-	-	-	-	-	-	-	-	-	-	-	-	-	-	-	-	-	-	-
38	1290	Thymol	Terpenes	Thyme	-	-	-	-	-	-	-	-	-	-	-	-	-	-	-	-	-	-	-	-	-	-	-	-	-	-	-	-	-	-	-	-	-	+	-	-	-	-	-	-
39	1291	Indole	Heterocyclic compounds	Burns	-	-	-	-	-	-	-	-	-	-	-	-	-	-	-	-	+	-	+	+	-	+	-	-	-	-	-	-	-	-	-	-	-	-	-	-	-	+	-	-
40	1338	Eugenol	Esters	Spice	+	+	-	-	+	-	-	+	-	-	-	-	-	+	-	+	-	-	-	+	+	-	+	+	+	+	+	+	+	-	-	+	+	+	-	+	+	+	+	+
41	1385	Beta-damascenone	Ketones	Floral	-	-	-	-	-	-	-	-	-	-	-	-	-	-	-	-	-	-	+	-	-	-	-	-	-	-	-	-	-	-	-	-	-	-	-	-	-	-	-	-
42	1400	Tetradecane	Alkanes	Alkanes	-	-	-	-	-	-	-	-	-	-	-	-	-	-	-	-	-	-	-	-	-	-	-	-	-	-	-	-	-	-	-	+	-	-	-	-	-	-	-	-
43	1417	Apha-ionone	Ketones	Wood	-	-	-	-	-	-	-	-	-	-	+	+	-	-	-	-	-	-	-	-	-	-	-	-	-	-	-	-	-	-	-	-	-	-	-	-	-	-	-	-
44	1421	Difurfuryl sulfide	Sulfides	Earthy	-	-	-	-	-	-	-	-	-	-	-	-	-	-	-	-	-	-	-	-	-	-	-	-	-	-	-	-	-	-	-	-	-	+	-	-	-	-	+	-
45	1479	2-tridecanone	Ketones	Herbal	-	-	-	-	-	-	-	-	-	+	-	+	+	+	-	-	-	-	-	-	-	-	-	-	-	-	+	-	+	+	+	+	-	-	-	-	-	-	-	-
46	1500	Beta-ionone	Ketones	Wood	-	-	-	-	-	-	-	-	-	-	-	-	-	+	-	-	-	-	-	-	-	-	-	-	-	-	-	-	-	-	+	-	+	+	-	-	-	+	-	-
47	1576	12-methyltridecanal	Aldehyde	Fruity	-	-	-	-	-	-	-	-	-	-	-	-	-	+	-	-	-	-	-	-	-	-	-	-	-	-	-	-	-	-	-	-	-	-	-	-	-	-	-	-
48	1070	Acetophenone	Ketones	Floral	-	-	-	+	-	+	-	-	-	+	-	-	-	-	-	-	-	-	-	-	-	-	-	-	-	-	-	-	-	-	-	-	+	-	-	+	-	-	+	+

Note: 1– non-sprayed, 2 – water, 3– phenylalanine 100 mg L^{-1}, 4– phenylalanine 200 mg L^{-1}, 5– tryptophan 100 mg L^{-1}, 6– tryptophan 200 mg L^{-1}, 7– tyrosine 100 mg L^{-1}, 8– tyrosine 200 mg L^{-1}.
